# Emerging Perspectives of RNA *N*^6^-methyladenosine (m^6^A) Modification on Immunity and Autoimmune Diseases

**DOI:** 10.3389/fimmu.2021.630358

**Published:** 2021-03-05

**Authors:** Lipeng Tang, Xingyan Wei, Tong Li, Yi Chen, Zhenhua Dai, Chuanjian Lu, Guangjuan Zheng

**Affiliations:** ^1^Department of Pharmacology of Traditional Chinese Medicine, The Second Clinical College of Guangzhou University of Chinese Medicine, Guangzhou, China; ^2^Department of Pathogen Biology, The Chinses Center for Disease Control and Prevention, Beijing, China; ^3^Department of Pharmacy, The Second Clinical College of Guangzhou University of Chinese Medicine, Guangzhou, China; ^4^School of Pharmaceutical Sciences, Guangzhou University of Chinese Medicine, Guangzhou, China; ^5^Section of Immunology, Guangdong Provincial Academy of Chinese Medical Sciences, Guangzhou, China; ^6^Department of Dermatology, The Second Affiliated Hospital of Guangzhou University of Chinese Medicine, Guangzhou, China; ^7^Department of Pathology, The Second Clinical College of Guangzhou University of Chinese Medicine, Guangzhou, China

**Keywords:** *N*^6^-methyladenosine (m^6^A), innate immunity, adaptive immunity, autoimmune disease, viral infection

## Abstract

*N*^6^-methyladenosine (m^6^A) modification, the addition of a methylation decoration at the position of N6 of adenosine, is one of the most prevalent modifications among the over 100 known chemical modifications of RNA. Numerous studies have recently characterized that RNA m^6^A modification functions as a critical post-transcriptional regulator of gene expression through modulating various aspects of RNA metabolism. In this review, we will illustrate the current perspectives on the biological process of m^6^A methylation. Then we will further summarize the vital modulatory effects of m^6^A modification on immunity, viral infection, and autoinflammatory disorders. Recent studies suggest that m^6^A decoration plays an important role in immunity, viral infection, and autoimmune diseases, thereby providing promising biomarkers and therapeutic targets for viral infection and autoimmune disorders.

## Introduction

*N*^6^-methyladenosine (m^6^A) modification, one of the most prevalent RNA chemical modifications, refers to the methylation of adenosine A at the position of N6. Despite the fact that the m^6^A modification of RNA was identified in the 1970s (1), the activation mechanism and biological function of m^6^A modification remained unclear until recently. Thanks to the advances in high-throughput sequencing technology, two research groups independently and simultaneously used methyl-RNA-immunoprecipitation-sequencing (MeRIP-seq) or m^6^A-seq to exploit the distribution and function of m^6^A modification in human genome during 2012, leading to better global methods for studying the RNA m^6^A methylation (2, 3). From then on, the biological and pathological functions of m^6^A have been extensively studied. Here we will highlight recent advances in understanding the biological process of m^6^A modification. Then we will discuss the critical functions of m^6^A modification in innate/adaptive immune responses, immune system development, and viral infection. Finally, we will address the contributory effects of m^6^A methylation on various autoinflammation or autoimmune diseases.

## The Dynamic and Reversible Process of m^6^A Modification

**Figure 1:**
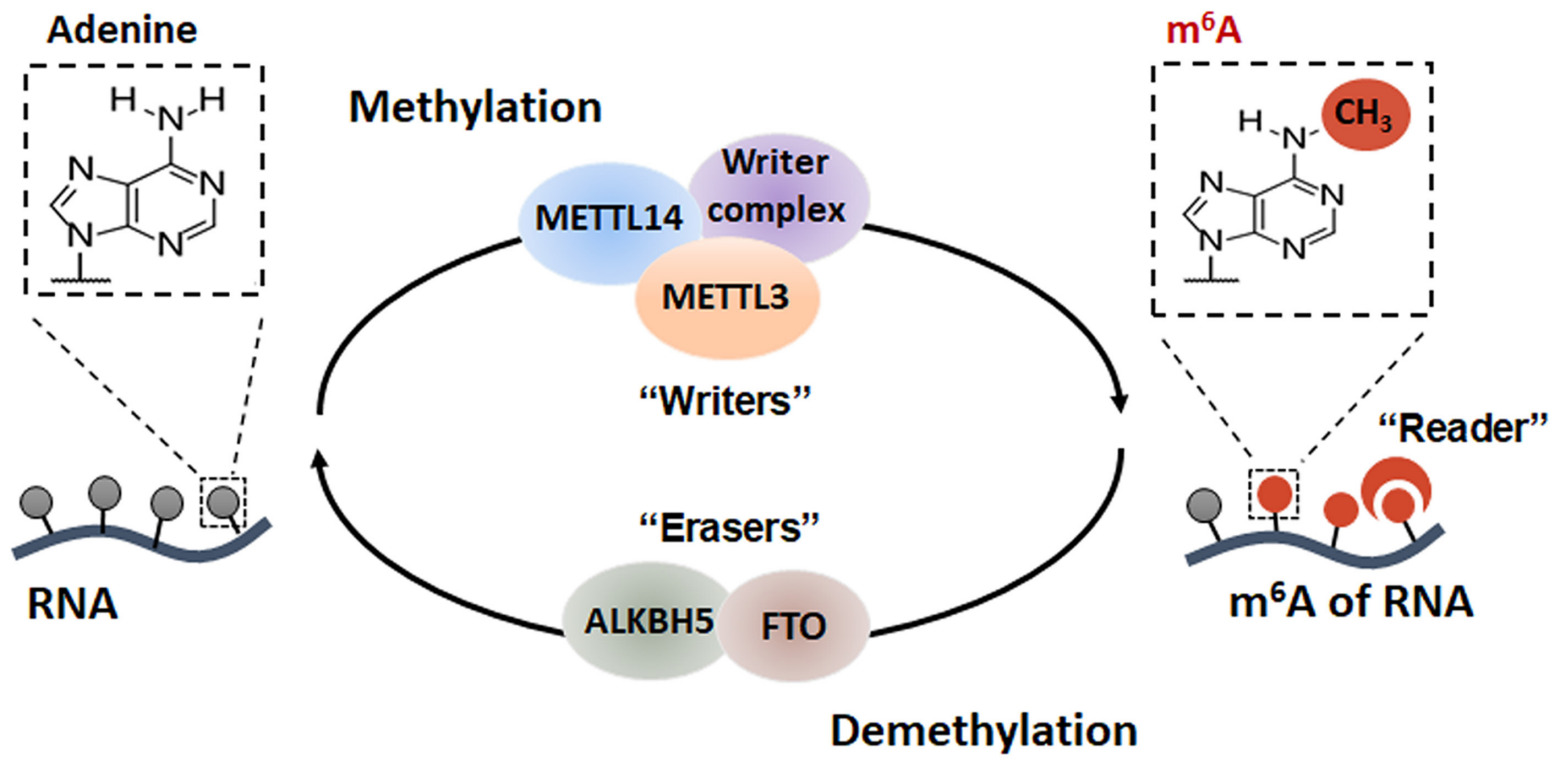
The dynamic methylation process of RNA m^6^A modification. The m^6^A decoration can be dynamically regulated by several m^6^A methyltransferases and demethylase.

The modification of m^6^A is dynamic and reversible, because it can be catalyzed by methyltransferase (also known as the m^6^A “writers”) or removed by demethylase (also known as the m^6^A “erasers”). The recognition of m^6^A modification is mediated by several nuclear and cytoplasmic RNA binding proteins (RBPs), including YTH-domain containing family proteins, insulin-like growth factor 2 mRNA-binding proteins (IGF2BPs) and heterogeneous nuclear ribonucleoproteins (HNRNPs) (Figure 1).

### m^6^A Writers

The decoration of m^6^A is mediated by a big methyltransferase complex consisting of Methyltransferase-like 3 (METTL3), METTL14, RNA binding motif protein 15 (RBM15), Wilms tumor 1 associated protein (WTAP), HAKAI (also known as Casitas B-lineage lymphoma-transforming sequence-like protein 1, CBLL1), zinc finger CCCH domain-containing protein 13 (ZC3H13), and Vir-like M6A Methyltransferase Associated (VIRMA, also known as KIAA1429) subunits. METTL3 is a predominant catalytic subunit of the writer complex, while METTL14 functions as an allosteric activator to enhance the catalytic activity of METTL3 (4). WTAP, a regulatory component of this complex, can bind to the target RNA and then recruit the METTL3 and METTL14 to form the catalytic core components (METTL3/METTL14/WTAP) that install the m^6^A methylation onto the target RNA (5). RBM15, which is an interacting partner of WTAP, serves as an adapter protein recruiting the m^6^A methyltransferase complex to U-rich regions (6). VIRMA, another important subunit of the writer complex, also plays an important role in guiding m^6^A modification in 3′ untranslated regions (3′ UTRs) and near stop codon (7). HAKAI acts synergistically with the other core components of the methyltransferase complex to form the m^6^A methylation (8). ZC3H13 exerts vital effects on anchoring and mediating nuclear m^6^A modification (9). This methylation writer complex usually installs the m^6^A modification on a specific and consensus RNA sequence, RRACH (R = G or A; H = U, A or C). Additionally, the m^6^A methylation is found to be enriched in 3′ UTRs, long exons, and near stop codons.

### m^6^A Erasers

The m^6^A decoration can be removed by demethylase, such as fat mass and obesity-associated protein (FTO) and AlkB homolog 5 (ALKBH5). FTO, which belongs to the non-heme Fe (II)- and α-KG-dependent dioxygenase AlkB family of proteins, has been identified as the first demethylase of m^6^A modification by Chuan He and his colleagues in 2011 (10). This study provided the first evidence that RNA m^6^A modification is reversible and dynamic, which is similar to the processes of methylation of DNA and histones. ALKBH5, which was identified to be another m^6^A demethylase in 2013, binds to specific m^6^A-modified single-stranded RNA, thereby catalyzing the demethylation of m^6^A (11).

### m^6^A Readers

**Figure 2:**
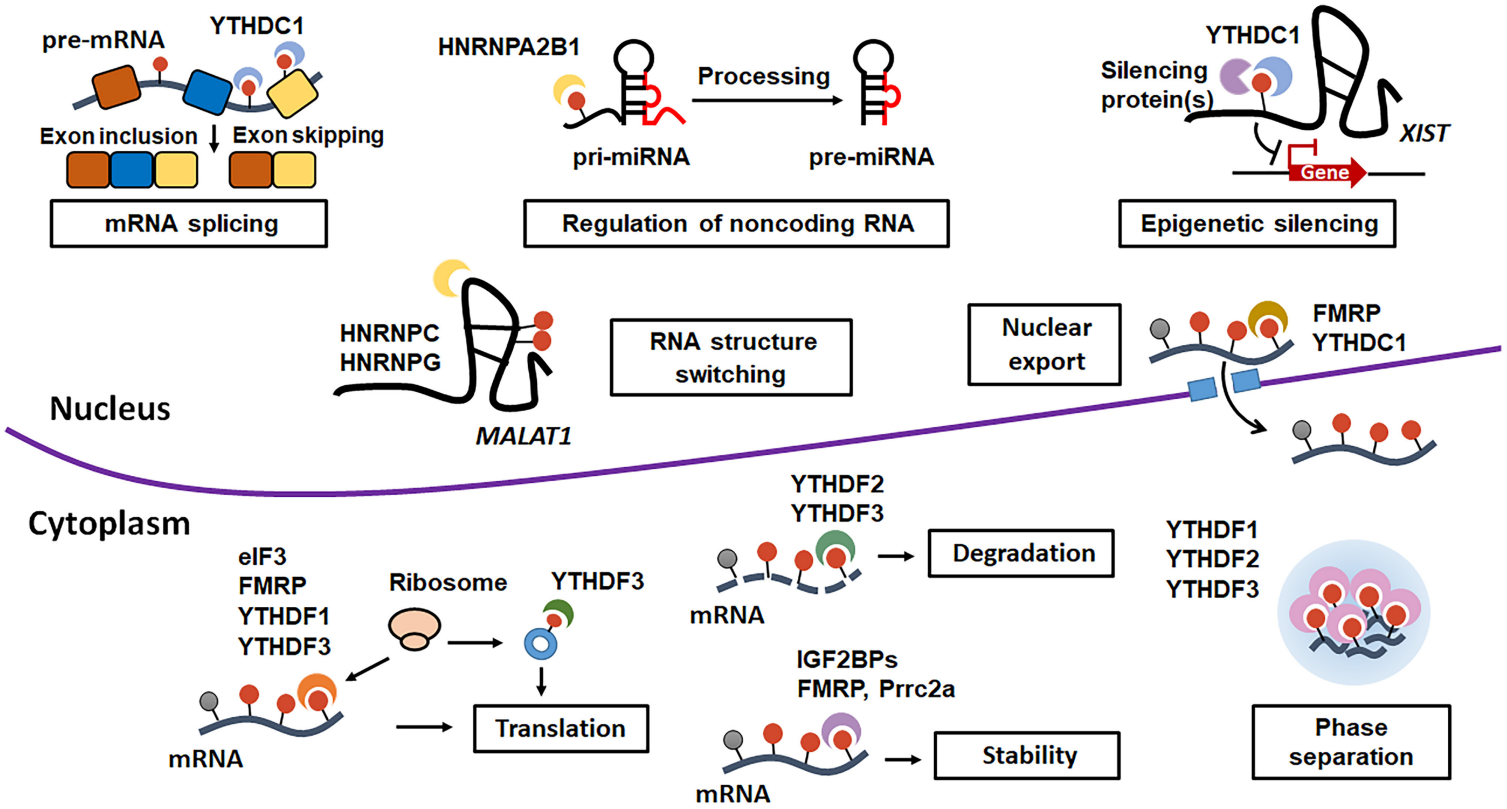
The diverse biological functions of m^6^A methylation exerted by different m^6^A-binding proteins. The nuclear m^6^A modification regulates mRNA splicing, RNA structure switching, epigenetic silencing, non-coding RNA modulation and nuclear export of RNA, while the cytoplasmic m^6^A methylation modulates RNA stability, RNA translation, and phase separation.

RNA-binding proteins, which can bind to m^6^A decoration and execute m^6^A-mediated biological functions, are referred to as m^6^A readers. According to their location, the m^6^A-binding proteins can be further divided into nuclear and cytoplasmic m^6^A readers: ① The nuclear m^6^A readers, including YTH domain containing 1-2 (YTHDC1-2), HNRNPA2B1 HNRNPC, HNRNPG, and Fragile X mental retardation protein (FMRP), play multiple roles in modulating mRNA splicing (12), epigenetic silencing (6), nuclear export of mRNA (13), regulation of non-coding RNA (14), and RNA structure switching (15); ② The cytoplasmic m^6^A readers, such as YTHDC2, YTH domain family 1-3 (YTHDF1-3), IGF2BPs, FMRP, and Proline rich coiled-coil 2 A (Prrc2a), exert regulatory effects on mRNA stability (16), mRNA translation (17), mRNA degradation (18–20), and phase separation (21, 22) (Figure 2).

## The m^6^A-Mediated Biological Functions

Emerging evidence has shown that the decoration of m^6^A and m^6^A-binding proteins regulates various post-transcriptional aspects of the mRNA lifecycle (Figure 2).

The nuclear m^6^A readers can affect several nuclear processes of RNA (mRNA and non-coding RNA) metabolism. For example, Xu et al. found that METTL3-mediated m^6^A decoration contributes to spermatogenesis through regulating the alternative splicing of spermatogenesis-associated genes (23). In addition, Pan et al. and Chang et al. found that m^6^A modification alters the secondary structure of mRNA and long non-coding RNA, ultimately increasing the binding of RNA and RBPs (15, 23, 24). They named this m^6^A-mediated RNA structural remodeling that enhanced RNA–RBPs interactions as “m^6^A-switch” (15). During 2016, P. Patil et al. further demonstrated that m^6^A methylation promotes long non-coding RNA X-inactive specific transcript (*XIST*)-mediated epigenetic gene silencing by stabilizing the silencing proteins on *XIST* (6). Additionally, growing evidence indicates that m^6^A readers promote nuclear export of mRNA. Roundtree et al. found that m^6^A reader YTHDC1 facilitates the export of m^6^A-modified mRNAs through interaction with serine and arginine rich splicing factor 3 (SRSF3)—nuclear RNA export factor 1 (NXF1) protein complexes (13). FMRP, another m^6^A reader, can modulate nuclear export of m^6^A-decorated mRNAs via interacting with nuclear RNA export factors (NXF2) or chromosomal maintenance 1 (CRM1) (25, 26). Moreover, Sohail F. Tavazoie's group demonstrated that m^6^A decoration acts as an important post-transcriptional regulator for miRNA biogenesis. They found that DGCR8 can recognize and efficiently process METTL3-methylated pri-miRNA (27). Further evidence indicated that the reorganization of m^6^A mark by DGCR8 is mediated by HNRNPA2B1 (14). Collectively, these studies indicate that m^6^A modification is a critical regulatory factor for RNA splicing, structure remodeling, epigenetic silencing, nuclear export, and non-coding RNAs regulation in the nucleus.

On the other hand, the cytosolic m^6^A readers also modulate many cytoplasmic processes of RNA metabolism. One of the most established functions of m^6^A is degradation of RNA. Compelling evidence demonstrated that m^6^A-methylated mRNAs can be degraded through YTHDF2/YTHDF3-mediated degradative pathway (18–20). Mechanistically, YTHDF2 directly recruits the CCR4-NOT deadenylase complex to its target transcripts, thereby resulting in degradation of m^6^A-containing mRNA through CCR4/NOT-induced deadenylation. Additionally, m^6^A-methylated mRNAs can be also decayed by YTHDF2-Heat-responsive protein 12 (HRSP12)-RNase P/MRP-mediated endoribonucleolytic cleavage (20). Moreover, YTHDF3 can induce m^6^A-decorated mRNAs deadenylation and sequent decay by recruiting PAN2-PAN3 deadenylase complex (28). Nonetheless, accumulating evidence also indicate that several m^6^A-readers, including IGF2BPs, FMRP and Prrc2a, can conversely enhance stability of m^6^A-containg mRNA (29–31). These studies provide valuable insights into the complex role of m^6^A modification in modulating mRNA stability and degradation. Interestingly, mounting evidence also demonstrates that m^6^A modification plays a contributory role in translation through distinct cellular mechanisms. One regulatory mechanism is that YTHDF1 interacts with eIF3 and consequently recruits the ribosomes to m^6^A-modified mRNAs to promote their translation (32). Another modulatory mechanism is that m^6^A modification within the 5′ UTR of mRNAs enhances translation via directly interacting with eIF3 but not cap-binding factor eIF4E, especially upon heat shock stress (33, 34). In addition, cytoplasmic METTL3 can directly associate with eIF3 subunit h (eIF3h) independent of its catalytic activity and ultimately induce translation of various oncogenic mRNAs (35, 36). Furthermore, m^6^A modification contributes to circRNAs-regulated extensive translation through recruiting initiation factor eIF4G2 and YTHDF3 (37). Remarkably, three independent research group simultaneously revealed that m^6^A RNA methylation enhances phase separation. They showed that poly-methylated mRNA can function as a scaffold that recruits and juxtaposes several m^6^A-binding proteins, such as YTHDF1, YTHDF2, and YTHDF3, finally forming liquid droplets composed of m^6^A-modified mRNA–YTHDF complexes and resulting in phase separation (21, 22, 38). Together, these studies provide the important experimental evidence that m^6^A modification plays a key role in regulating multiple cytoplasmic biological processes, such mRNA stability or destabilization, mRNA translation, and phase separation.

## The Key Role of m^6^A Modification in the Immune System

### The Role of m^6^A Modification in Innate Immune System

The innate immunity is an arm of the immune system that comprises the cells and mechanisms that provide the first line of defense against infections in a non-specific manner. Innate immune cells, such as monocytes, macrophages, neutrophils, natural killer cells, can sense invading pathogens (such as virus and bacteria) and exogenous RNAs and subsequently respond to pathogens infections. Additionally, dendritic cells (DCs), which function as messengers between the innate and the adaptive immune systems, can process antigen materials from pathogens and present them on the cell surface to activate T cells. Accumulative evidence indicates that m^6^A modification and m^6^A-related proteins participate in innate immunity via regulating the recognitions of and responses to unmodified transcribed RNA (tRNA), invading pathogens, exogenous RNAs and aberrant endogenous double-stranded RNA (dsRNA). Additionally, m^6^A-methylation also plays a complex role in the DCs activation and functions.

**Figure 3:**
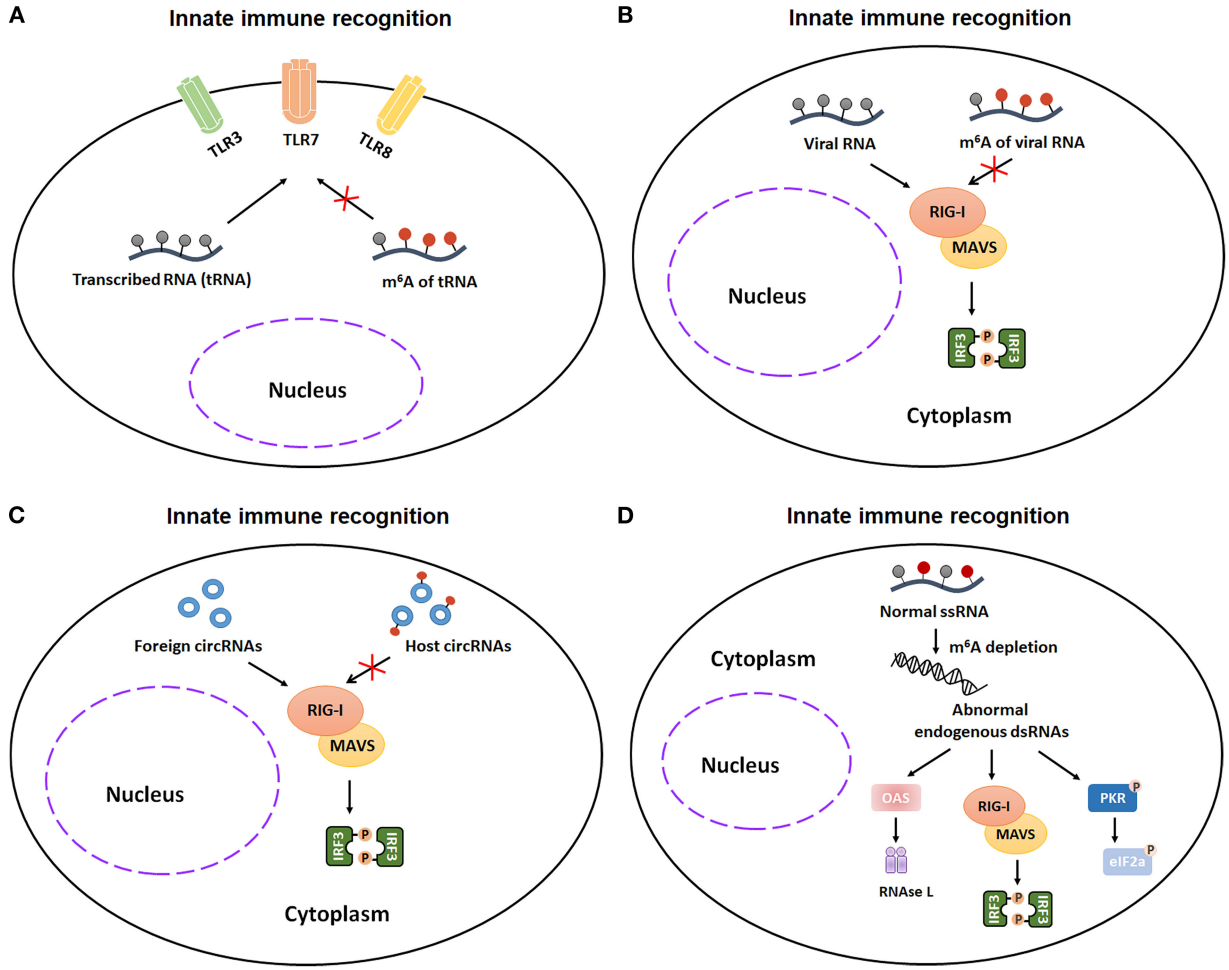
m^6^A modification participates in innate immunity via regulating the recognitions of unmodified transcribed RNA (tRNA), invading pathogens, exogenous RNAs, and aberrant endogenous double-stranded RNA (dsRNA). **(A)** m^6^A-modified tRNA reduces or eliminates the TLR3, TLR7, or TLR8 activation in monocyte-derived DCs (MDDCs). **(B)** m^6^A-methylation favors virion RNA escaping from the innate immunity recognitions in Huh7 cells and A549 cells. **(C)** Exogenous circRNAs, which lack m^6^A modifications, can be recognized by RIG-I signaling pathway in Hela cells. **(D)** Aberrant endogenous dsRNA in hematopoietic stem cells, which is formed by loss of m^6^A addition in ssRNA, can be recognized by RIG-I as foreign dsRNA and subsequently trigger a deleterious innate immune response. TLR3, Toll-like receptor 3; TLR7, Toll-like receptor 7; TLR8, Toll-like receptor 8; tRNA, transcribed RNA; RIG-I, retinoic acid-inducible gene I; MAVS, mitochondrial antiviral signaling protein; IRF3, interferon regulatory transcription factor 3; ssRNA, single-stranded RNA; dsRNA, double-stranded RNA; OAS, 2',5'-oligoadenylate synthetase gene; PKR, protein kinase R.

Recognition of invading pathogens is the initial step of innate immunity. The detection of invading pathogens is dependent on a number of pattern-recognition receptors, including plasma membrane receptors (Toll-like receptors, TLRs) and cytosolic sensor (RIG-I-like receptors, RIG-I and NLR proteins). During 2005, Karikó et al. firstly demonstrated that nucleoside modifications, such as m^5^C, m^5^U, Ψ, s2U, and m^6^A modification, reduce or eliminate the TLR3, TLR7, or TLR8 activation in monocyte-derived DCs (MDDCs) (39). This finding represents the first indication of an interferential effect of m^6^A modification on the process of RNA recognition. As m^6^A modification is also widely distributed in viral mRNA, it's possible that viruses use m^6^A-modification to evade host immune recognition. In line with this speculation, Jianrong Li and Lee Gehrke demonstrated that m^6^A-methylated virion RNA, such as HMPV (human metapneumovirus) and HCV (hepatitis C virus), bound RIG-I with a very low affinity, finally disturbing the conformational change of RIG-I and reducing the sequent RIG-I-mediated innate immune responses in A549 cells and Huh7 cells (40, 41). These discoveries further proved that viruses can take advantage of m^6^A-modification as camouflage to escape from host immune system. Similar to linear RNAs, circRNAs, a class of loop non-coding RNAs, can also utilize m^6^A-modification to avoid immune detection. Endogenous circRNAs, in which introns transfer and deposit the m^6^A modifications onto the exons before their final back-splicing, can bypass host immune recognition. However, synthetic circRNAs, which lack m^6^A modifications, can be discriminated by RIG-I in Hela cells (42). Moreover, endogenous ssRNA (single-stranded RNA) with m^6^A modifications can prevent abnormal immune recognition and keep the innate immune response at steady state. Gao et al. demonstrated that endogenous ssRNA without m^6^A modifications in hematopoietic stem cells can be recognized by RIG-I as foreign dsRNA and subsequently trigger a deleterious innate immune response and hematopoietic failure (43). Taken together, these studies demonstrate that the m^6^A modification in tRNA, viral RNA, host circRNAs, and normal endogenous ssRNA can serve as a critical factor to allow innate immune system to avoid abnormal immune recognition (Figures 3A–D).

**Figure 4:**
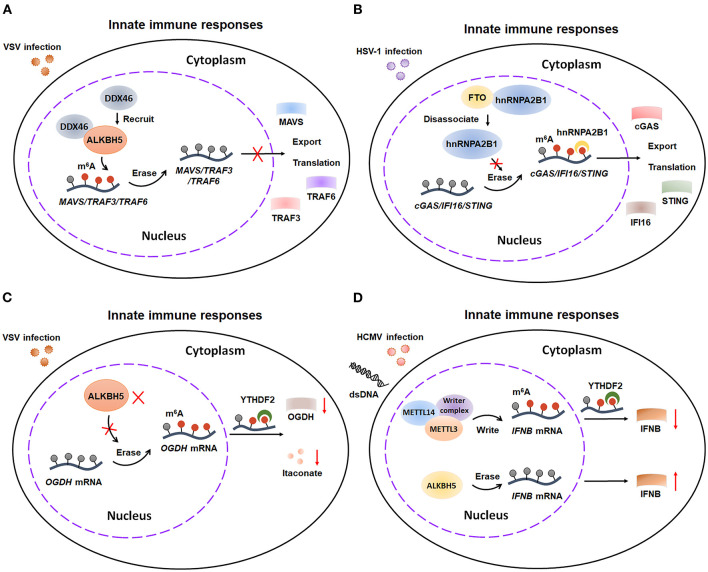
m^6^A modification is involved in antiviral responses of innate immunity. **(A)** DDX46, which forms a complex with ALKBH5, inhibits antiviral responses by reducing m^6^A modification-mediated nucleus export and translation of *MAVS, TRAF3*, and *TRAF6* transcripts. **(B)** hnRNPA2B1 enhances antiviral responses by preventing FTO-mediated demethylation on *STING, CGAS*, and *IFI16* transcripts. **(C)** During VSV infection, the enzymatic activity of ALKBH5 is decreased, which results in increasing m^6^A level. The upregulation of m^6^A modification leads to decreasing the expression of OGDH transcripts and reprograming host cell metabolism, which consequently enhancing antiviral responses. **(D)** m^6^A modification suppresses antiviral responses by repressing the production and stability of *IFNB* mRNA stability. VSV, vesicular stomatitis virus; TRAF3, TNF receptor-associated factor 3; TRAF6, TNF receptor-associated factor 6; HSV-1, herpes simplex virus-1; STING, stimulator of interferon response CGAMP interactor 1; CGAS, cyclic GMP-AMP synthase; IFI16, interferon gamma inducible protein 16; OGDH, α-ketoglutarate dehydrogenase; HCMV, Human cytomegalovirus; dsDNA, double stranded DNA; IFNB, IFN-beta.

Once RNA recognition happens, innate immunity is immediately activated and releases multiple cytokines, such as type I interferons (IFNs) (44, 45). The role of m^6^A modifications in innate immune response is much more complex and controversial. Several researches illustrated that m^6^A modifications contributes to innate defense against viral infection (Figures 4A–C). The host innate responses will be suppressed by erasing the m^6^A modification from several antiviral transcripts. Mechanistically, DDX46, which forms a complex with ALKBH5, inhibits antiviral innate responses against VSV infection by reducing m^6^A modification-mediated nucleus export and translation of *MAVS/TRAF3/TRAF6* (46). Consistently, impeding demethylation can amplify the innate immune responses. Wang et al. reported that hnRNPA2B1 enhances m^6^A-dependent nucleocytoplasmic trafficking and translation of *STING, CGAS*, and *IFI16* mRNAs by preventing FTO-mediated demethylation, ultimately leading to amplifying the host innate immune response to HSV-1 infection (47). Moreover, m^6^A modifications-mediated cellular metabolic reprogram facilitates host immunity against viral infection. Mechanistically, the enzymatic activity of ALKBH5 is decreased upon viral infection, which consequently increases m^6^A modifications on the mRNA of α-ketoglutarate dehydrogenase (OGDH). The m^6^A modifications then downregulate the OGDH and its downstream metabolite itaconate, finally reprograming host cell metabolism and suppressing viral replication. Thus, in these studies, m^6^A modifications exerts contributory effects on antiviral responses through increasing export and translation of some key antiviral components and rewiring cellular metabolism. However, Noam Stern-Ginossar's and Ian Mohr's researches illustrated that m^6^A modifications plays an obstructive role in innate immunity response (44, 45) (Figure 4D). METTL3/METTL14-mediated m^6^A modification decreases the production and stability of *IFNB* mRNA, eventually promoting HCMV replication *in vitro* and *in vivo*. In contrast, demethylation by ALKBH5 promotes the production of *IFNB* mRNA, ultimately elevating type I interferon level. These results indicate that m^6^A modification acts as a negative modulator of type I interferon-mediated antiviral responses. Future studies are warranted to further exploit the precise role of m^6^A modification in innate immune responses.

**Figure 5:**
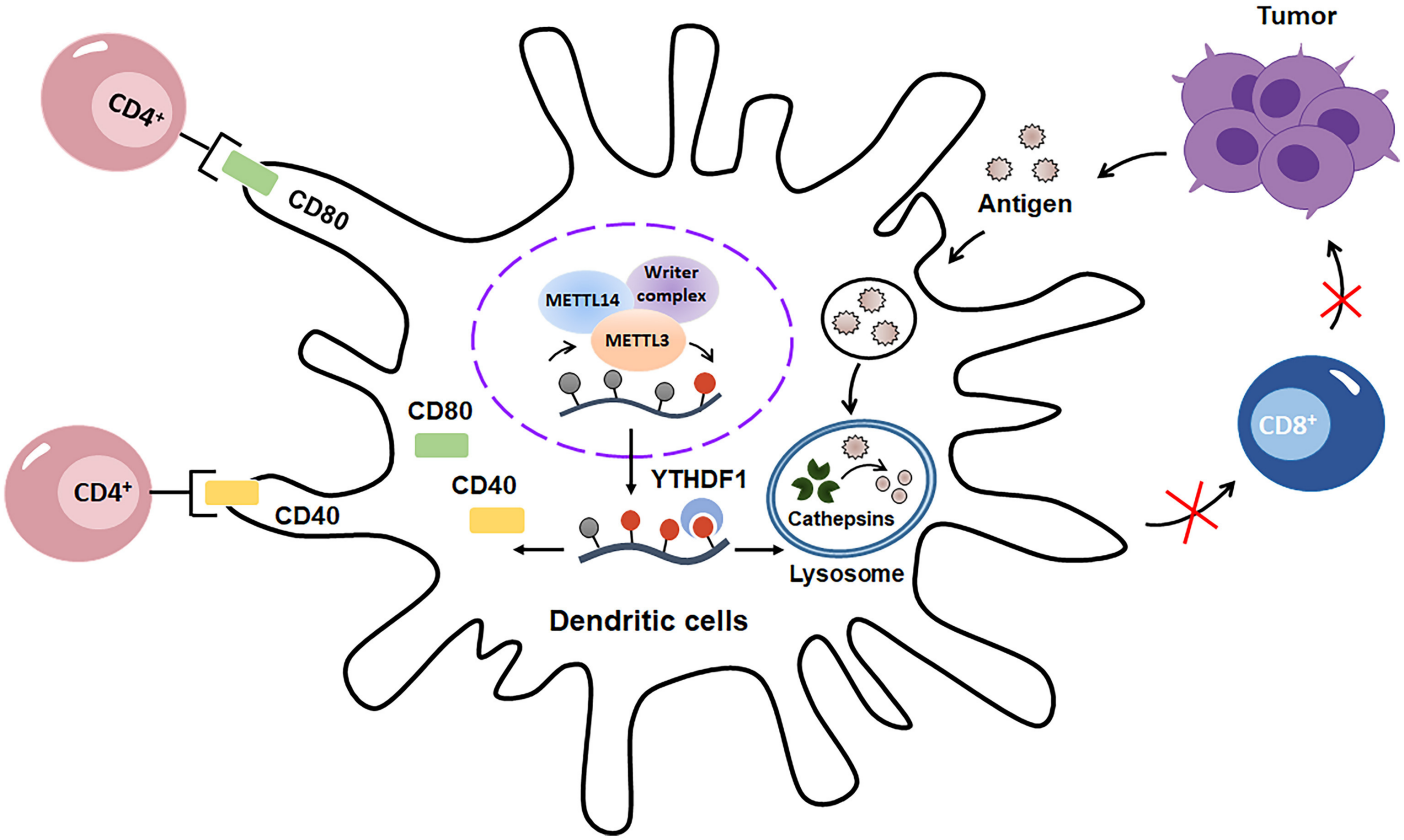
m^6^A modification has double-sided regulatory effects on dendritic cells (DCs). On one hand, m^6^A methylation promotes DCs maturation and activation through enhancing the translation of CD40, CD80. On the other hand, m^6^A modification decreases the antigen-presentation capacity of DCs by increasing the translation of cathepsins in lysosomes, which consequently decays the tumor antigens and suppress sequent CD8^+^-associated anti-tumor immunity.

Mounting evidence has shown that m^6^A methylation is critical for DCs activation and functions (Figure 5). On one hand, m^6^A methylation promotes DCs maturation. Huamin et al. showed that METTL3-mediated mRNA m^6^A methylation is essential for dendritic cell (DC) maturation and function (48). Mechanistically, METTL3 enhances the translation of CD40, CD80, and TLR4 signaling adaptor Tirap in DCs through an m^6^A catalytic activity-dependent manner, ultimately promoting DC maturation and strengthening TLR4/NF-κB signaling pathway signaling during DC activation and DC-mediated T cells–priming (Figure 5). On the other hand, m^6^A methylation may decrease the antigen-presentation capacity of DCs. Dali and colleagues demonstrated that the cross-presentation of tumor antigens and the cross-priming capacity of tumor-associated DCs are enhanced in *Ythdf1*^−/−^ mice. Moreover, the CD8^+^ T cell-mediated tumor antigens-specific immune response is elevated in *Ythdf1*^−/−^ mice owing to the enhancement of cross-presentation of DCs (49). Mechanistically, m^6^A-binding protein YTHDF1 can elevate the translation of lysosomal cathepsins in DCs, which consequently degrade the tumor antigens and suppress their antigen-presentation as well as CD8^+^ T cells-priming ability (Figure 5). Taken together, these studies indicated that m^6^A modification may play dual roles in enhancing or suppressing DCs activation and function in a context dependent way. Further studies are needed to expand our understanding and uncover the potential role of m^6^A methylation in DCs maturation, activation and function in future.

### The Role of m^6^A Methylation in Adaptive Immune System

**Figure 6:**
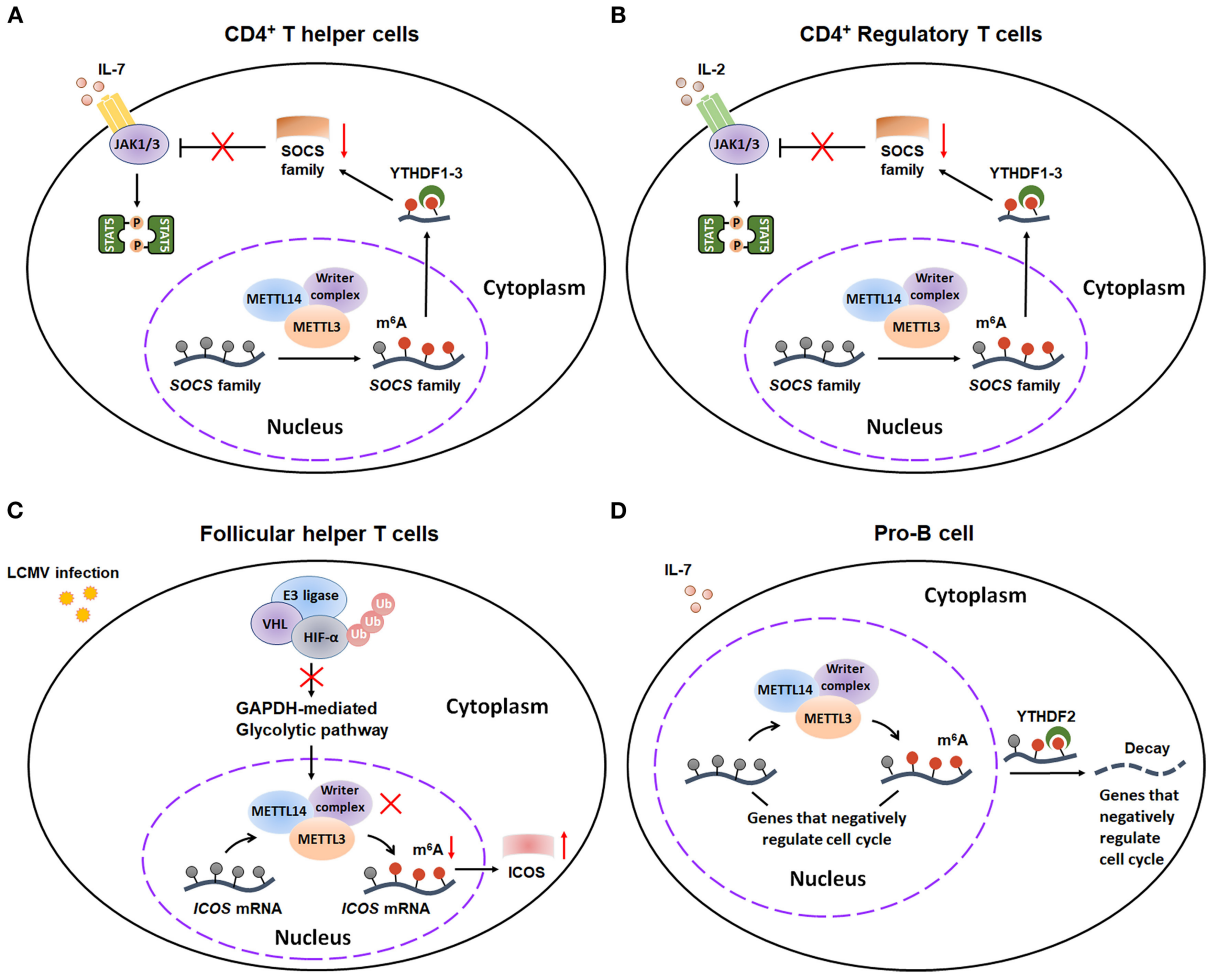
m^6^A methylation plays a critical role in adaptive immunity. **(A)** m^6^A modification can promote the CD4^+^ T helper cells proliferation and differentiation by decaying *SOCS* mRNAs and consequently activating IL-7-STAT5 pathway. **(B)** m^6^A methylation plays an essential role in sustaining the suppressive functions of CD4^+^ T Regulator cells by decaying *SOCS* mRNAs and consequently activating IL-2-STAT5 pathway. **(C)** E3 ligase VHL promotes the early initiation of Tfh cells development via suppressing HIF-1α-GAPDH–mediated glycolysis, ultimately enhancing ICOS expression by reducing m^6^A-labed *ICOS* mRNA. **(D)** METTL14-mediated m^6^A modification facilitates IL-7-induced pro-B cell proliferation by decreasing a group of transcripts that negatively regulate cell cycle. SOCS, suppressor of cytokine signaling; JAK, Janus Kinase; STAT5, Signal transducer and activator of transcription 5; VHL, Von Hippel–Lindau; HIF-1α, hypoxia-inducible factor 1α; ICOS, inducible T cell costimulatory; Ub, Ubiquitination.

The adaptive immunity is another arm of the immune system that specializes in the clearance of specific pathogens. It is mediated by the activation of antigen-specific T/B lymphocytes, ultimately establishing long-lasting immunological memory against the given antigen. Emerging evidence indicates that m^6^A exerts a vital effect on adaptive immunity (Figure 6).

m^6^A modification serves as a critical modulator for the differentiation and function of different subsets of T cells, including CD4^+^ T helper cells (Th1 and Th17 cells), CD4^+^ Regulatory T cells (Treg cells) and Follicular helper T cells (Tfh cells). By using CD4-Cre conditional *Mettl3*^lox/lox^ mice, Richard Flavell's group found that m^6^A modification controls T cell homeostatic expansion, especially affecting the proliferation and differentiation of CD4^+^ Th1 and Th17 cells (50). Mechanistically, m^6^A modification has an inducible degradative effect on *SOCS* mRNAs, consequently promoting T cell homeostatic proliferation and differentiation via increasing IL-7-mediated STAT5 activation (Figure 6A). Additionally, Richard A Flavell, Hua-Bing Li, and coworkers gave another evidence that m^6^A mRNA methylation is also essential for sustaining the suppressive functions of Treg (51). Similarly, m^6^A decoration also plays an important role in degradation of *SOCS* mRNAs, thus activating IL-2-STAT5 signaling pathway to maintain the suppressive functions and cell stability of Treg cells (Figure 6B). On contrary, m^6^A mRNA methylation might exert an inhibitory effect on the Tfh cells differentiation (52). Recently, Yun-Cai Liu et al. illustrated that E3 ligase Von Hippel–Lindau (VHL) promotes the early initiation of Tfh cells development via suppressing hypoxia-inducible factor 1α (HIF-1α)—mediated glycolysis. Mechanistically, VHL deficiency results in activation of HIF-1α-GAPDH glycolytic pathway and consequently reduction in ICOS (inducible costimulator) expression by enhancing m^6^A modification on *ICOS* mRNA, ultimately leading to attenuated Tfh cell differentiation (Figure 6C). Taken together, these studies extend our understanding of crucial regulatory effects of m^6^A decoration on the differentiation of T cells.

Additionally, m^6^A also has essential effects on early B cell development. By using *Mb1*^cre/+^*Mettl14*^fl/fl^ mice, Haochu Huang et al. found that METTL14 deficiency dramatically reduced the B cell numbers (53). Then the researchers further divided the CD19^+^B220^mid^Ig^k/l^ population into pro-B cells, early/late large pre-B cells, and small pre-B cells. Further study indicated that loss of METTL14 led to inhibition of the pro-B cell proliferation and large-to-small-pre-B transition. Mechanistical study demonstrated that METTL14 promotes B cell development through two distinct ways: ① METTL14-mediated m^6^A modification that facilitates IL-7-triggered pro-B cell proliferation by decreasing a series of YTHDF2-bound m^6^A-labeled mRNA (Figure 6D); ② The large- to small-pre-B transition, which is independent of m^6^A-regconized proteins YTHDF1/2. Actually, this transition is largely dependent on the METTL14-mediated proper transcriptional activation of several critical transcription factors (TFs). Collectively, this finding represents the first indication that m^6^A modification participates in the development of B cells. However, future studies are needed to further explore the mechanism by which METTL14 regulates the transcriptional activation of these key TFs.

## m^6^A Modification in Viral Infection

Despite the critical roles of m^6^A decoration in host anti-viral responses, emerging evidence also indicate that m^6^A modification might play a vital role in viral lifecycle and infection. In fact, the presence of m^6^A addition in several viruses, such as Influenza A virus (IAV) (54), B77 avian sarcoma virus (55), and Rous Sarcoma Virus (56), has been reported several decades ago. However, the potential regulatory effects of m^6^A modification on viral life cycle remains unclear until recently.

### Human Immunodeficiency Virus 1 (HIV-1)

**Figure 7:**
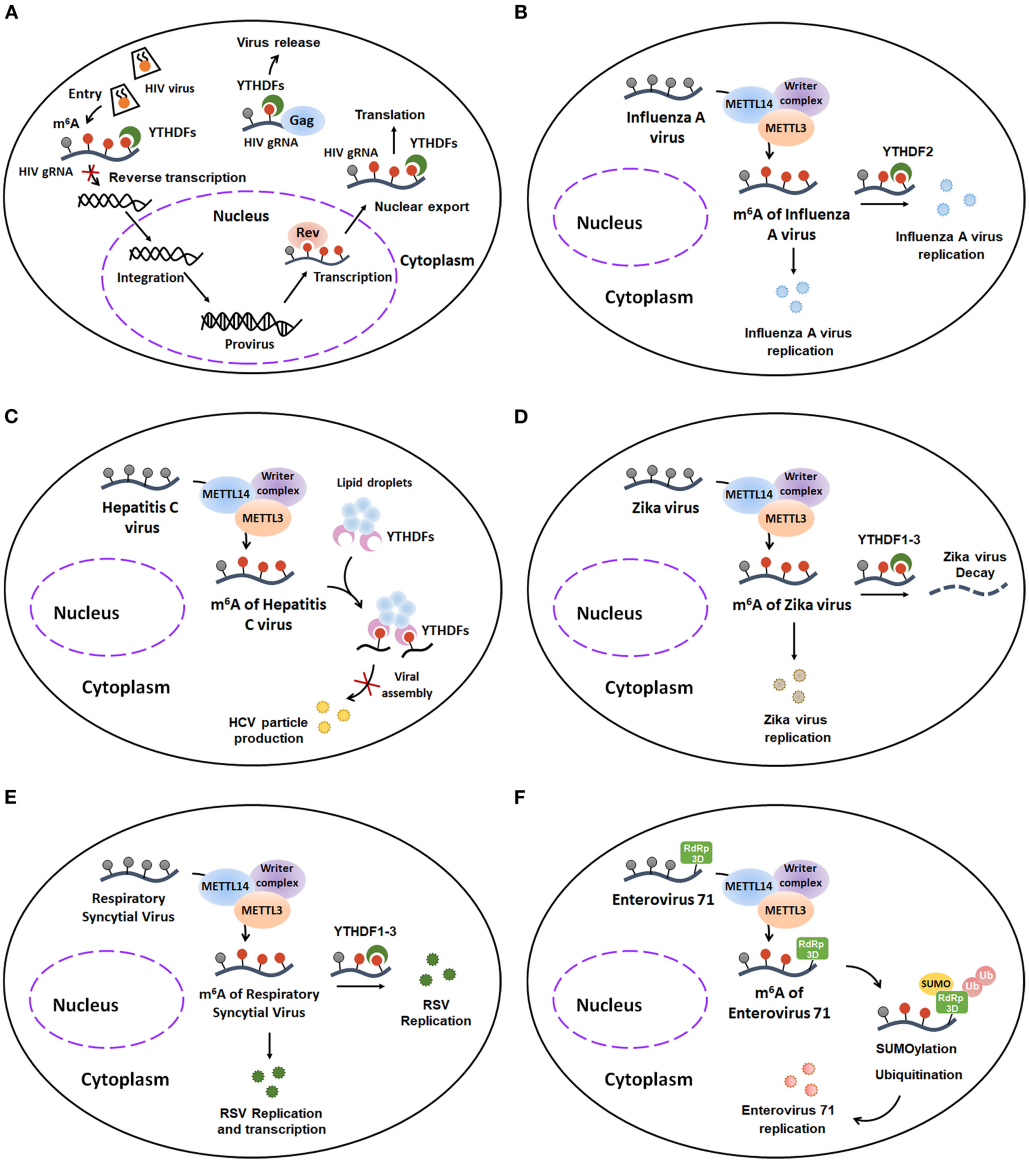
The host m^6^A machinery regulates viral RNA metabolism during viral infection. **(A)** m^6^A modification plays a complex role in HIV infection. Early upon entry, YTHDF1–3 proteins bind to m^6^A-modified HIV-1 NL4.3 Genome and induce degradation of the HIV gRNA, finally impeding reverse transcription of HIV-1. Once integrated, m^6^A modification favors the Rev-mediated nuclear export of HIV RNA and YTHDF1–3 regulated translation of HIV-related protein. Moreover, YTHDF1–3 can form a complex with HIV-1 Gag and viral RNAs, culminating in an enhancement of progeny HIV-1 particles release and an inhibition of progeny HIV-1 virus infectivity. **(B)** m^6^A addition contributes to Influenza A virus expression and replication. **(C)** m^6^A decoration negatively regulates HCV particle production by interrupting with viral assembly. **(D)** m^6^A installation promotes Zika virus replication. However, the m^6^A-labeled Zika virus RNA can be degraded when it binds to YTHDF1-3 proteins. **(E)** m^6^A methylation enhances the replication and transcription of Respiratory Syncytial Virus. **(F)** m^6^A modification facilitates Enterovirus type 71 replication by co-localizing with and further enhancing the SUMOylation and K63-linked ubiquitination of EV71 RNA-dependent RNA polymerase 3D (RdRp 3D) protein. HIV, Human immunodeficiency virus 1; HIV gRNA, HIV genomic RNA; HCV, Hepatitis C virus; RSV, Respiratory Syncytial Virus; RdRp 3D protein, RNA-dependent RNA polymerase 3D protein; SUMO, SUMOylation; Ub, Ubiquitination.

Human immunodeficiency virus 1 (HIV-1), a lentivirus belonging to the Retroviridae family, can cause acquired immunodeficiency syndrome (AIDS). Several studies tried to identify the potential role of m^6^A modification in HIV infection. Firstly, Lichinchi et al. found that the m^6^A levels of host and viral mRNAs are dramatically increased in MT4 T-cells during HIV-1 LAI strain infection. In addition, this m^6^A installation is critical for replication and nuclear export of HIV-1 (57). Depletion of METTL3/METTL14 inhibits the HIV replication by inducing a decrease in *gp120* mRNA and CAp24 protein levels, whereas depletion of ALKBH5 results in enhanced HIV replication with a remarkable increase of the HIV-1 *env* gene. Moreover, researchers found that two m^6^A-labeled adenosines (A7883 and A7877) within the stem loop II region of HIV-1 *Rev response element (RRE)* RNA can promote the interaction between *RRE* and HIV-1 Rev protein, finally enhancing the nuclear export of HIV RNA. Later Kennedy et al. identified four to six m^6^A clusters in the 3′ UTR region of HIV-1 NL4.3 Genome in human CD4^+^ CEMSS T-cells by using photo-crosslinking-assisted m^6^A sequencing (PA-m^6^A-seq) techniques. Additionally, they demonstrated that the YTHDF1-3 proteins, especially YTHDF2, can bind to these m^6^A decoration and consequently increase the expression of HIV-1 genomic RNA (gRNA) and p24 capsid protein, ultimately enhancing virus proteins translation at 48 h post-infection (hpi) (58). However, there is contradictory evidence regarding whether viral m^6^A modification positively regulates HIV replication or not. In the contrast with Kennedy's results, Chuan He and Li Wu's group indicated that YTHDF1–3 proteins, which can bind to m^6^A-modified HIV-1 NL4.3 genome in HeLa/CD4^+^ T cells, suppress HIV-1 post-entry infection via degrading HIV-1 gRNA and inhibiting both early and late viral reverse transcription at 24 h post-infection (hpi) (59). Moreover, Chuan He and Li Wu's groups recently confirmed that YTHDF1-3 can bind to two m^6^A-modified GGACU motifs (positions 198 and positions 242) within 5′ UTR of HIV-1 NL4.3 gRNA and then form a complex with HIV-1 Gag and viral RNAs, resulting in an enhanced release of HIV-1 particles. However, these progeny HIV-1 viruses have lower infectivity (60). Collectively, these studies suggest that m^6^A modification may have a complex function on HIV life cycle in the host cells: ① Upon early entry, YTHDF1–3 proteins bind to m^6^A-modified HIV-1 NL4.3 genome and induce degradation of the HIV gRNA, finally impeding reverse transcription of HIV-1; ②Once integrated, m^6^A modification favors the Rev-mediated nuclear export of HIV RNA and YTHDF1–3 regulated translation of HIV-related protein; ③ Moreover, YTHDF1–3 can form a complex with HIV-1 Gag and viral RNAs, culminating in an enhancement of progeny HIV-1 particles release and an inhibition of progeny HIV-1 viruses infectivity (Figure 7A).

### Influenza A Virus (IAV)

Influenza A virus (IAV), an enveloped negative-sense RNA virus belonging to the Orthomyxoviridae family, is one of the major etiologic agent of human respiratory tract infections and might result in severe infection and even death. Although previous studies have identified that IAV contains numerous m^6^A modification (61), the specific functions of m^6^A addition in IAV are poorly understood. Recently, Courtney *et al*. further demonstrated that m^6^A modifications preferentially deposit on the IAV plus (mRNA) and minus (vRNA) strands which encode viral structural proteins, including HA, M, NP and NA. Additionally, m^6^A modification is critical for IAV gene expression and replication (62). They found that the gene expression and viral titer of IAV are dramatically reduced in the METTL3 knockout A549 cells, whereas the expression and virion production of IAV are obviously increased in A549 cells with overexpression of YTHDF2. To further clarify the role of m^6^A modification in regulating IAV pathogenicity *in vivo*, the authors generated IAV mutants with m^6^A site mutations on plus strand or minus strand of the hemagglutinin (HA) segment. They found that these m^6^A-deficient IAV mutants attenuate their pathogenicity in mice. Taken together, these results indicate that the m^6^A modification is a positive regulatory factor for IAV expression and replication *in vitro* and *in vivo* (Figure 7B).

### Hepatitis C Virus (HCV)

Hepatitis C virus, a small enveloped positive-sense single-stranded RNA virus of the Flaviviridae family, is the major cause of hepatitis C and hepatitis C-mediated liver cancer. Accumulating evidence indicates that the m^6^A modification plays a vital regulatory role in life cycle of HCV (Figure 7C). Gokhale et al. demonstrated that m^6^A addition, which is installed within HCV during its infection, can negatively regulate HCV particle production (63). The production of HCV RNA and viral titer were enhanced after METTL3 and METTL14 depletion. Conversely, depletion of FTO reduced the expression of HCV RNA and the viral titer of infectious HCV. Consistently, HCV m^6^A mutants within the viral E1 gene increased the HCV particle production. Mechanistically, the m^6^A-mediated decrease of HCV particle production was caused by the m^6^A-binding YTHDF proteins. During HCV infection, the cytosolic YTHDF proteins re-localize to lipid droplets and then directly interact with m^6^A-labeled HCV mRNA through DRA^m^CH motif. The interaction of YTHDF proteins and HCV mRNA leads to the abrogation of binding between Core protein and HCV RNA genomes, ultimately resulting in interrupting with viral assembly and suppressing HCV particle production. In summary, these studies indicate that m^6^A installation exerts a negative effect on HCV particle production.

### Zika Virus

Zika virus (ZIKV), another enveloped positive-sense single-stranded RNA virus of the Flaviviridae family, typically causes fever, rash, headache, conjunctivitis in adults, and microcephaly in infants. Recently, the relevance of ZIKV to m^6^A modification is reported by Chuan He and Tariq M Rana's research groups (64). Using methylated RNA immunoprecipitation sequencing (MeRIP-seq), they discovered that there are 12 m^6^A peaks in the full-length of ZIKV RNA. Furthermore, they illustrated that the m^6^A modifications promote ZIKV replication. In addition, YTHDF family proteins, especially YTHDF2, bind directly to and further degrade ZIKV RNA. Interestingly, ZIKV infection also changes the m^6^A deposition and methylation motifs of host RNA. Taken together, unlike HCV, m^6^A decoration positively regulates Zika virus replication (Figure 7D).

### Respiratory Syncytial Virus (RSV)

Respiratory Syncytial Virus (RSV), which belongs to non-segmented negative-sense (NNS) RNA viruses, is a leading cause of respiratory disease in infants, the elderly and immunocompromised individuals. To date, there is only one research article illustrating the relevance of RSV to m^6^A addition. Xue et al. demonstrated that m^6^A nucleosides are abundant in both RSV genome and antigenome. In addition, RSV can use the host m^6^A machinery to methylate itself. Interestingly, the m^6^A modification exerts promotive effects on RSV genome replication, gene expression and virus production (65). They found that the gene expression and viral titer of RSV are significantly decreased when m^6^A writer proteins, such as METTL3 and METTL14, are knocked down. In contrast, RSV replication is notably increased upon knockdown of m^6^A eraser proteins. Additionally, m^6^A reader proteins YTHDF1-3, especially YTHDF2, can directly bind to RSV RNAs and subsequently enhance replication and transcription of RSV. Furthermore, recombinant RSVs with abrogation of m^6^A sites in G gene and G mRNA are defective in replication in cultured human airway epithelial (HAE) cells and in respiratory tracts of cotton rats. Together, these findings reveal the pro-viral effects of m^6^A modification on RSV replication, transcription, and particle production, suggesting that targeting m^6^A modification may be a good way to develop antiviral drugs or vaccine for RSV (Figure 7E).

### Enterovirus 71 (EV71)

Enterovirus type 71 (EV71), a non-enveloped single-stranded RNA virus belonging to the Picornaviridae family, is one of the main pathogens that causes hand-foot-and-mouth disease (HFMD) in infants and young children. The relevance between EV71 and m^6^A modification is poorly known. Recently, using UHPLC-MS/MS, Hao et al. confirmed that EV71 RNA contains m^6^A modification. In addition, the host m^6^A machinery can install the m^6^A modifications into the EV71 mRNA in the cytoplasm during EV71 infection. Furthermore, these m^6^A decorations are critical for EV71 replication. The viral titer and the viral RNA copy numbers of EV71 are significantly decreased in the METTL3-deficient Vero cells, whereas these inhibitory effects can be reserved when the demethylases FTO is knocked down. Additionally, the replication of EV71 is suppressed when the EV71 RNA contains m^6^A site mutations at 3,056 ~ 4,556 NT in C residues. Mechanistically, METTL3 facilitates EV71 replication by co-localizing with and further enhancing the SUMOylation and K63-linked ubiquitination of EV71 RNA-dependent RNA polymerase 3D (RdRp 3D) protein (66). Collectively, these studies suggest that m^6^A decoration contributes to EV71 replication (Figure 7F). Targeting m^6^A addition might be a good therapeutic measure to treat the EV71 infection.

## m^6^A Modification in Autoimmune Diseases

### Systemic Lupus Erythematosus

Systemic lupus erythematosus (SLE) is a typical multi system autoinflammatory disorder characterized by the presence of pathogenic autoantibodies and immune complex deposition in skin, joints, kidneys, and serosal membranes (67). Previous studies demonstrated that epigenetic changes, such as histone modification and DNA methylation, participate in the progression of SLE (68, 69). However, the role of RNA modification in SLE remained unclear until recently. Firstly, Li et al. mentioned a potential link between m^6^A modification and SLE in a comprehensive review (70). Then, Luo et al. found that the mRNA expression levels of METTL14?ALKBH5 and YTHDF2 were significantly decreased in peripheral blood mononuclear cells (PBMCs) of SLE patients (71, 72). Importantly, downregulation of ALKBH5 and YTHDF2 was found to be risk factors for SLE after multivariate logistic regression analysis. However, it still lacks direct mechanistical data to confirm the precise role of m^6^A methylation in the pathogenesis of SLE. Future studies are needed to further explore the potential effect of m^6^A decoration on SLE and underlying mechanisms.

### Rheumatoid Arthritis

Rheumatoid arthritis (RA) is a systemic and disabling autoimmune disorder characterized by chronic and progressive joint erosion. The main pathological features of RA are synovial inflammation, cartilage injury, and bone erosion (73). Previous studies indicated that genetic and epigenetic factors contribute to the pathogenesis of RA. However, the exact cause of RA is poorly known. Using large scale Genome-Wide Association Studies (GWAS), Mo et al. identified 37 RA-associated m^6^A-SNPs in Asian or European populations (74). To figure out the underlying mechanisms of these RA-associated m^6^A-SNPs, researchers further investigated the associations of m^6^A-SNPs with the expression of local genes. Finally, they formed 23 SNP-Gene-RA trios after integrating the m^6^A-SNPs and local gene expression data. However, more experimental studies are required to further confirm whether these RA-associated m^6^A-SNPs are really involved in the pathogenesis of RA. Recently, Wang et al. reported that METTL3 is significantly elevated in the PBMCs of RA patients. Interestingly, the upregulation of METTL3 is positively associated with the level of CRP (C-reactive protein) and ESR (Erythrocyte sedimentation rate) in serum, indicating that the level of METTL3 in PBMCs could be used to predict the disease activity of RA. Mechanistically, upregulated METTL3 by LPS can trigger pTHP-1 macrophages activation and subsequent inflammatory response through NF-κB signaling pathway (75). This study illustrates that upregulation of METTL3 expression in macrophage may contribute to RA. However, it's still unknown whether METTL3-mediated m^6^A addition in macrophage is implicated in the pathogenesis of RA or not. Further studies are warranted to fully classify the potential role of m^6^A modification in RA.

### Multiple Sclerosis

Multiple sclerosis (MS), a chronic immune-regulated disease of the central nervous system (CNS), is characterized by various CNS lesions, including demyelination, neurodegeneration, optic neuritis, and chronic axonal damage (76). Growing evidence suggests that genetic changes may result in MS. However, the key susceptibility genes of MS are largely unknown. Using GWAS and integrating m6AVar database, Xing-Bo and coworkers found that rs2288481 (p.Glu183Lys) in DKKL1 gene and rs923829 in METTL21B might be novel susceptibility loci for MS. Furthermore, these authors validated the association of rs923829~METTL21B and rs2288481~DKKL1 in the PBMCs of 40 unrelated Chinese Han individuals. They found that rs923829 is significantly associated with METTL21B expression levels, whereas the association between rs2288481 and DKKL1 is not statistically significantly (77). This research represents the first effort to illustrate the potential causal effects of m^6^A-related proteins on MS. However, we still lack the clear evidence that the dysfunctional m^6^A modification directly participates in the pathogenesis of MS. Future studies should firstly detect the level of m^6^A modification in MS and further elucidate its modulatory effect and underlying mechanism on MS.

### Inflammatory Bowel Disease

Inflammatory bowel disease (IBD), including Crohn's disease and ulcerative colitis, is characterized by chronic relapsing intestinal inflammation. Although the etiology of IBD is ill-defined, the dysregulation of proinflammatory Th17 cells and anti-inflammatory Treg cells contributes to the pathogenesis of IBD (78, 79). Using CD4-Cre^+/*Tg*^ Mettl14^*FL*/*FL*^ conditional knockout mice, Thomas X et al. firstly demonstrated that METTL14 deficiency in T cells causes the development of spontaneous colitis by dramatically increasing the release of Th17 cell-related proinflammatory cytokines (80). Given the important role of m^6^A modification in maintaining Treg cell stability and their suppressive functions, it is reasonable to find that deletion of METTL14 impairs induction of Treg cells and consequently results in the imbalance between Th17 and Treg cells, ultimately inducing spontaneous colitis development. This study provides novel insights into recognizing the pathogenesis of IBD. Further studies are warranted to prove whether targeting m^6^A addition is a good therapeutic approach to the treatment of IBD.

## Potential Diagnostic and Therapeutic Application of m^6^A Modification

Since virus utilizes m^6^A methylation as a strategy to mark as “self” RNA and evade the detection by innate immunity system (41), targeting viral m^6^A decoration might be a good measure to detect viral infection and to enhance innate immune responses to viruses.

Given the stability of circRNAs, they can be developed as an effective strategy to deliver agents. However, as mentioned above, delivery of exogenous circRNAs might induce circRNA immunogenicity *in vivo*. Therefore, researchers should be very careful when they use exogenous circRNA as vehicles for gene delivery. On the other side, the synthetic circRNAs-induced immunogenicity can also be exploited to induce anti-tumor immunity (42).

The m^6^A modification does open a new door for anti-tumor immunity. Besides the synthetic circRNAs-mediated anti-tumor Immunity, targeting FTO also contributes to anti-tumor Immunity. Su et al. found that inhibition of FTO by genetic depletion or pharmacological antagonist can sensitizes acute myeloid leukemia (AML) cells to T cell cytotoxicity by suppressing Leukocyte Immunoglobulin Like Receptor B4 (LILRB4) (81). However, there is also conflicting evidence about the role of m^6^A methylation in anti-tumor immunity. As described above, m^6^A decoration represses the antigen-presentation capacity of DCs by promoting the expression of lysosomal proteases, finally reducing the antigen-specific anti-tumor immunity (49). Taken together, these studies indicate that m^6^A modification may be a double-edged sword in anti-tumor immune response. More studies will be needed to further illustrate the precise role and the underlying mechanism of m^6^A methylation in anti-cancer immunity.

The identification of the potential contributory role of m^6^A modification in several autoimmune diseases is of great significance to develop effective targeted therapeutics. However, the current knowledge of the effects of m^6^A methylation on autoinflammatory disorders is still in its infancy. A better understanding of the promotive effects of m^6^A decoration on the development of autoimmune disorders is warranted.

## Conclusions and Future Perspectives

Our understanding of the biological and pathological functions of m^6^A modification has rapidly progressed due to the advances in the m^6^A-related high-throughput sequencing technology. These novel findings demonstrate that RNA m^6^A methylation can function as an important post-transcriptional regulator that modulates innate/adaptive immunity responses. In addition, aberrant expression of m^6^A modification may play a contributory role in the pathogenesis of autoimmune diseases.

As we know, the RNA m^6^A methylation is dynamic. It will be very interesting to investigate the real expression level of m^6^A modification in certain physiological or pathological contexts. Taking advantage of complete loss-of-function mutants of m^6^A writers, erasers and readers may illustrate how the m^6^A machinery proteins regulate immune responses as well as immune-related diseases. We can use high-throughput sequencing technologies to forecast the potential methylation sites (82). Nevertheless, the deposition specificity of m^6^A methylation is still poorly understood. Further studies are needed to investigate the mechanism underlying the modification specificity of m^6^A methylation.

Although FTO was firstly identified as the first RNA demethylase of m^6^A modification, emerging controversial studies report that FTO also plays an important role in m^6^A_m_ and m^1^A demethylation. Recently, Wei et al. demonstrated that the subcellular location of FTO, which is different between distinct cells, determines the differential modulatory pattern of FTO demethylation (83). Nuclear FTO prefers to mediate internal m^6^A demethylation in polyadenylated mRNA, m^6^A in U6 RNA, internal and cap m^6^A_m_ in small nuclear RNAs (snRNAs) and m^1^A demethylation in tRNA, whereas cytoplasmic FTO preferentially regulates internal m^6^A and cap m^6^A_m_ demethylation in polyadenylated mRNA as well as m^1^A demethylation in tRNA. Collectively, these studies indicate that FTO likely mediates m^6^A, m^6^A_m_, and m^1^A demethylation through a spatial regulatory spectrum.

Pre-mRNA alternative splicing, a nuclear process including precise removement of introns and joining of exons, is critical for generating multiple transcript isoforms from one single gene. During alternative splicing, precise excision of introns can result in exon inclusion, whereas excision of introns and skipping of internal exons lead to exon skipping (Figure 2). Accumulative evidence has demonstrated that m^6^A modification acts as a key factor that regulates pre-mRNA exon inclusion or exon skipping splicing in the nucleus. For example, Xiao and coworkers illustrated that YTHDC1 selectively recruits pre-mRNA splicing factor SRSF3, which promotes exon inclusion, but blocks binding of exon-skipping SRSF10 to targeted mRNAs, ultimately resulting in alternative splicing of targeted transcripts (12). To illustrate how m^6^A addition regulates the pre-mRNA splicing, Yang et al. proposed a m^6^A-mediated alternative splicing model: (1) m^6^A decoration in exonic regions mainly results in exon inclusion; (2) m^6^A modification in intronic regions could lead to both either exon inclusion or exon skipping (84). Further studies are needed to confirm whether the different prevalence and distribution of m^6^A methylation in either exonic regions or intronic regions really determine the subsequent splicing mode.

Although several studies have indicated that HNRNPA2B1 can recognize the m^6^A modification and then execute m^6^A-associated biological functions, the specific binding pattern between HNRNPA2B1 and m^6^A decoration is poorly known. Recently, the structural and biochemical results have shown that hnRNP A2/B1 may function as an indirect m^6^A “reader.” Wu et al. reported that hnRNP A2/B1 does not specifically recognize m^6^A-labeled RNA (85). Neither full-length hnRNP A2/B1 nor tandem RRMs has a higher binding affinity to m^6^A-containing RNA than non-methylated RNA *in vitro*. Additionally, these researchers indicated that very few m^6^A sites exhibit proximal hnRNP A2/B1 binding *in vivo*. Taken together, these studies suggest that hnRNP A2/B1 may execute m^6^A-related biological functions indirectly.

Previously, the different m^6^A readers were studied separately to figure out their specific or distinct functions. A series of researches demonstrated that some m^6^A readers, which even belong to the same protein family, may exert opposite effects. For example, YTHDF1 is reported to enhance the translation of targeted RNA (86, 87). By contrast, YTHDF2 promotes the decay of m^6^A-labed RNA (88, 89). Recently, Sara Zaccara and Samie R. Jaffrey proposed a new unified model for YTHDFs mediated-m^6^A-depedent-functions (90). In contrast to the previous model that different DF paralogs recognize different m^6^A binding-sites, they demonstrated that all three DF paralogs can bind to the same m^6^A binding-sites. They found that all three YTHDFs proteins have the same m^6^A-binding structures, equivalent m^6^A decoration affinities, similar protein binding partners and identical intracellular localizations. Additionally, they presented conflicting evidence that all DF paralogs preferentially interact with RNA degradation machinery. The effect of m^6^A-mediated degradation is most pronounced when all three YTHDFs proteins are available. They also found that YTHDF1 and YTHDF3 do not regulate the m^6^A-mRNAs translation after reanalysis of the DF1-related ribosome profiling (32) and the DF3-associated PAR-CLIP dataset (91). Moreover, they indicated that all three DF paralogs can work together to suppress m^6^A-mediated differentiation. Further evidence is needed to confirm whether YTHDF proteins just lead to RNA degradation in the future.

Since YTHDC1 also contains low-complexity domains (6), it's possible that YTHDC1 also has the ability to form phase separation. Future studies are needed to explore the potential role of YTHDC1 in phase separation, which might uncover a novel biological function of YTHDC1 in promoting phase separation. Additionally, Gokhale et al. observed that the cytosolic YTHDF proteins, which re-localize to lipid droplets during HCV infection, can bind to and consequently suppress the HCV particle production (63). These studies suggest that YTHDFs-mediated phase separation may play an important role in HCV infection.

m^6^A modification is a double-edged sword for host immunity and virus infection. On one side, virus can take advantage of m^6^A methylation to escape the recognition by innate immune cells. Additionally, several viruses, such as IAV and RSV, can utilize m^6^A modification for further replication in the host cell. On the other side, m^6^A modifications contributes to host innate defense against viral infection. It's will be very interesting to figure out the precise m^6^A-mediated crosstalk between virus and host cells. Moreover, specifically targeting viral m^6^A addition and promoting host m^6^A-mediated antiviral type I IFN response might be promising for the treatment of viral infection.

Interestingly, Liu et al. recently identified that SARS-CoV-2 (Severe Acute Respiratory Syndrome coronavirus 2), an enveloped RNA coronavirus causing a global health emergency, is gradually m^6^A methylated in 3′ end of the viral genome during its infection in the host cells. However, these m^6^A modifications negatively modulate SARS-CoV-2 life cycle. The viral replication is dramatically increased upon knockdown of METTL3 and METTL14 (92). This study suggests that m^6^A decoration may be a negative regulator for SARS-CoV-2 infection, providing a potential therapeutic avenue for the development of vaccine and antiviral drugs of SARS-CoV-2.

Given that m^6^A methylation plays a critical role in viral infection and autoinflammatory disorders, the identification of drugs targeting m^6^A modification is attractive. Using 3D proteome-wide scale screening, Malacrida et al. recently illustrated that the imidazobenzoxazin-5-thione (MV1035), a newly found sodium channel blocker, is an efficient ALKBH5 inhibitor *in vitro* (93). In parallel, Su *et al*. identified two compounds (CS1 and CS2) as FTO antagonist by docking and screening 260,000 compounds. The newly identified m^6^A modification regulators do open novel therapeutic avenues for understanding and treating m^6^A methylation-associated diseases. However, further studies are needed to confirm their efficacy and specificity *in vivo*.

## Author Contributions

LT, XW, and TL wrote the manuscript. XW, TL, and YC searched the literature. ZD, CL, and GZ edited the paper. All authors contributed to the article and approved the submitted version.

## Conflict of Interest

The authors declare that the research was conducted in the absence of any commercial or financial relationships that could be construed as a potential conflict of interest.
